# Vulcanizate Structures of SBR Compounds with Silica and Carbon Black Binary Filler Systems at Different Curing Temperatures

**DOI:** 10.3390/polym12102343

**Published:** 2020-10-13

**Authors:** Il Jin Kim, Donghyuk Kim, Byungkyu Ahn, Hyung Jae Lee, Hak Joo Kim, Wonho Kim

**Affiliations:** 1 Department of Polymer Science & Chemical Engineering, Pusan National University, Busandaehak-ro 63beon-gil, Geumjeong-gu, Busan 46241, Korea; tigerkim@hankooktech.com (I.J.K.); ehdgurzxc@gmail.com (D.K.); 2 Global Quality Management Team, Hankooktire & Technology Co., Ltd. HQ, 286 Pangyo-ro, Bundang-gu, Seongnam-si, Gyeonggi-do 13494, Korea; LHJ930@hankooktech.com (H.J.L.); hakjoo@hankooktech.com (H.J.K.); 3 Wet Braking Innovation TFT, Hankooktire & Technology Co., Ltd. R&D Center, 50, Yuseong-daero 935beon-gil, Yuseong-gu, Daejeon 34127, Korea; bkahn@hankooktech.com

**Keywords:** binary filler system, rubber compound, styrene-butadiene rubber, cure temperature, vulcanizate structures

## Abstract

The tire industry has shown an increasing demand for the reduction in rolling resistance. Efforts have been made to improve the viscoelastic properties of tire compounds and reduce the weight of tires through optimization of the vulcanizate structure, which has become extremely complex. In this study, vulcanizates using carbon black and silica as binary fillers were prepared at various curing temperatures. Vulcanizate structures with respect to curing temperature were classified according to the chemical crosslink density by sulfur, carbon black bound rubber (i.e., physical crosslink due to carbon black), and silica-silane–rubber network. All properties exhibited a decreasing trend under the application of high curing temperatures, and the decrease in the crosslink density per unit content of filler with an increase in curing temperature was shown to be greater in carbon black than in silica. Mechanical and viscoelastic properties were also measured to evaluate the impact that the compound variates have on tire tread performance. These results serve as a guideline for determining the content and filler type and for setting the cure condition during the design of actual compound formulations to increase the crosslink density of rubber while retaining the necessary mechanical and viscoelastic properties for practical application.

## 1. Introduction

The performance and physical properties of various rubber products are primarily determined by the type of rubber, reinforcing fillers, and the type and content of accelerators such as sulfur. In addition, the processing and curing conditions of rubber lead to changes in the performance and properties of rubber products. When vulcanization with sulfur was discovered by Goodyear, 8 wt% of sulfur with respect to rubber was used, and the corresponding cure time at 140 °C was 5 h. Since then, Oenslager discovered the accelerated reaction of sulfur with rubber due to aniline, which shortened the cure time, and continuous development of accelerators led to the development of guanidine [1]. In recent years, research on reducing the cure time of rubber has been actively pursued for technological development in response to a greater demand for productivity and diversification of manufacturing techniques. The most conventional method for reducing the cure time is through an increase in the cure temperature [2]. It is generally known that a higher cure temperature has the effect of shortening the cure time, but an increase in the cure temperature results in negative effects, such as a decrease of the mechanical properties and scorch safety, such that selecting the appropriate cure temperature is of vital importance [3,4].

With recent developments in the automotive industry, there has been an increasing demand for improving fuel efficiency, which has led to a continuously increasing demand for the reduction in the coefficient of rolling resistance (RRc) of tires. Developments on various polymers and on silica and silane as substitutes for carbon black reinforcing fillers have been performed to reduce RRc. In particular, vulcanized rubber filled with carbon black in tire treads shows excellent reinforcement of mechanical properties and enhancement of abrasion performance but faces drawbacks of increased heat generation. To overcome these weak points, a portion of the reinforcing filler is replaced with silica, resulting in improved tear strength and cut and chip resistance compared to using carbon black alone [5,6]. Currently, there are limitations to the use of silica and novel polymers for RRc reduction, and studies on reducing RRc through tire weight reduction are being conducted to overcome these challenges. In general, tire weight reduction is achieved by lowering the tread depth, and the core technique necessary for reducing the tread depth is improving the abrasion resistance of the tread compound. Therefore, it is possible to apply both silica and carbon black to simultaneously enhance the viscoelastic properties and achieve weight reduction by reducing the tread depth [7,8]. In addition to studies on altering these materials and tire structures, research on changing the cure condition is underway to improve the RRc performance of tires [9]. The cure condition is an important factor that affects the physical properties of the vulcanizate, and studies on the effect of temperature on the chemical structure of vulcanizates have been widely performed [10,11,12,13]. In general, lowering the cure temperature led to an increase in the number of poly-sulfide structures, which, in turn, improved the viscoelastic properties [14,15].

The major factors that determine the mechanical properties of rubber are the dispersion of the filler in the rubber, the interaction between the filler and rubber, and the vulcanizate structure caused by sulfur. In particular, the vulcanizate structure, expressed as the crosslink density, has the most significant effect on the mechanical properties of the rubber. In addition, the equilibrium swelling ratio in the organic solvent varies based on the density of the crosslinked network of the compound. The crosslink density of unfilled vulcanizate was formulated as an equation by Flory and Rehner in 1950 from the swelling phenomenon [16,17]. The Flory–Rehner equation is presented in Equation (1):

(1) $$\upsilon =\;\frac{1}{2M_{c}}=\;-\frac{\ln\left(1-V_{1}\right)+V_{1}+\chi V_{1}{}^{2}}{2\rho_{r}V_{0}\left(V_{1}{}^{\frac{1}{3}}-\frac{V_{1}}{2}\right)}$$

where *ν* is the crosslink density (mol/g), *M_c_* is the average molecular weight between the crosslink points (g/mol), *V*_1_ is the volume fraction of rubber in the swollen gel at equilibrium, *V*_0_ is the molar volume of the solvent (cm^3^/mol), *ρ_r_* is the density of the rubber sample (g/cm^3^), and *χ* is the polymer–solvent interaction parameter.

Kraus [18] and Boonstra [19] confirmed that adhering fillers such as carbon black in filled vulcanizate suppresses the swelling behavior and affects the crosslink density. The proportionality between such behavior and the volume fraction of the filler is expressed as the Kraus equation shown in Equation (2):

(2) $$\frac{v_{r0}}{v_{r}}=1-m\left(\frac{\varphi }{1\;-\;\varphi }\right)$$

where *ν_r_*_0_ is the volume fraction of rubber in the swollen unfilled rubber, *ν_r_* is the volume fraction of rubber in the swollen filled rubber, *m* is the slope corresponding to the filler–rubber interaction, and *φ* is the volume fraction of the filler.

**Figure 1:**
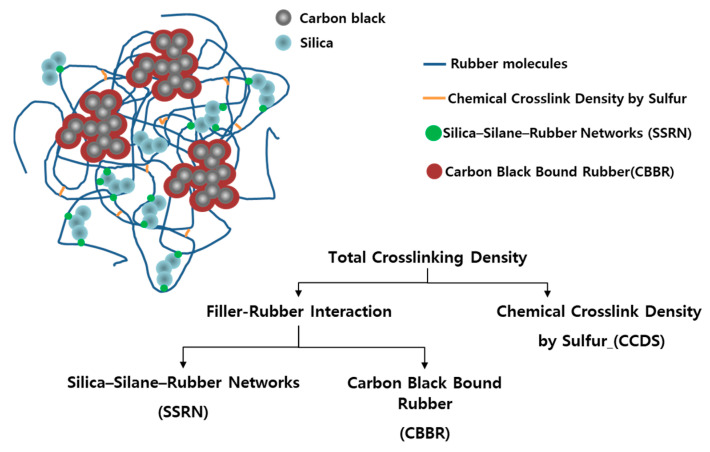
Vulcanizate structures with a carbon black and silica binary filler system.

In recent studies, vulcanizate structures in compounds using silica as the filler were classified quantitatively according to the network from silica-silane–rubber and the chemical crosslink by sulfur [20,21,22,23,24]. In addition, as shown in Figure 1, there are existing studies on compounds using carbon black and silica as binary fillers that are distinguished based on the chemical crosslink density by sulfur (CCDS), carbon black bound rubber (CBBR) (i.e., the physical crosslink due to carbon black), and silica-silane–rubber network (SSRN) [25]. As the filler–rubber network could not be subdivided prior to these research findings, it was difficult to characterize the detailed vulcanizate structure based on cure temperature and determine the effect of cure temperature on the physical properties of the material.

In this study, compounds were prepared by using styrene butadiene rubber (SBR) as the base polymer and carbon black and silica as binary fillers. Vulcanizates were prepared by varying the cure temperature, and the vulcanizate structures were categorized into CBBR, SSRN, and CCDS. Furthermore, the mechanical and viscoelastic properties that affect the performance of tire tread compounds were investigated to determine the correlation between the vulcanizate structure and the sensitivity to cure temperature. The research findings in this study are expected to serve as important factors during the design of compound formulations using a binary filler system.

## 2. Materials and Experimental Methods

### 2.1. Materials

Emulsion styrene butadiene rubber (ESBR, SBR1502, Kumho Petrochemical Co. Ltd., Seoul, South Korea; styrene content: 23.5 wt%) was used as the base rubber. Carbon black (N110, OCI Company Ltd., Seoul, South Korea; iodine adsorption: 135–145 g/kg, dibutyl phthalate absorption: 125–135 cm^3^/100 g), and silica (Ultrasil 7000 GR, Evonik Industries AG, Essen, Germany; Brunauer–Emmett–Teller surface area: 160–175 m^2^/g) were used as fillers. X50-S (Evonik Industries AG, Essen, Germany; silane 50%, carbon black N330 50%), which is a 1:1 mixture of carbon black and bis-[3-(triethoxysilyl)propyl]tetrasulfide (TESPT), was used as the silane coupling agent. Treated distillate aromatic extract (TDAE) oil (Kukdong Oil & Chemicals Co., Ltd., Yangsan, South Korea), ZnO, stearic acid (all from Sigma-Aldrich Corp., Seoul, Korea), *N*-(1,3-dimethylbutyl)-*N*′-phenyl-p-phenylenediamine (6PPD, Kumho Petrochemical Co., Ltd., Seoul, South Korea), and 2,2,4-trimethyl-1,2-dihydroquinoline (TMQ, Sinopec Corp., Beijing, China) were used as compounding ingredients. Sulfur (Daejung Chemicals & Metals Co., Ltd., Siheung, South Korea), *N*-tert-butyl-2-benzothiazyl sulfonamide (TBBS, Shandong Yanggu Huatai Chemical Co., Ltd., Liaocheng, China), and 1,3-diphenyl guanidine (DPG, Merck KGaA, Darmstadt, Germany) were used as curatives. *N*-(cyclohexylthio)phthalimide (Shandong Yanggu Huatai Chemical Co., Ltd., Liaocheng, China) was used as a pre-vulcanization inhibitor (PVI).

### 2.2. Preparation of Compound

In order to analyze the vulcanizate structure, the compounds were prepared with varying amounts of filler. A kneader (MSI-IRM 300, 300 cc, Mirae SI Co. Ltd., Gwangju, Korea) was used to manufacture the compounds according to the formulations shown in Table 1. The amount of TESPT was calculated as 8 wt% of the silica content. The amount of the silane coupling agent, X50-S, was calculated as 16% of the weight of silica because X50-S contained 50 wt% of TESPT. The 50% carbon black contained in X-50S was N330 grade with a large particle size. Unlike the small particle size N110 used in this experiment, X-50S was excluded when calculating the ratio of carbon black and silica. The compounds were mixed in the kneader for 10 min 40 s at a fill factor of 70%. The initial temperatures of the mixer for the first and second stages were 110 °C and 50 °C, respectively. The dump temperatures were controlled at 150–155 °C and 100 °C, respectively. In the first stage, both the silica and silane coupling agents were added with proportions of half and half. This was done to prevent the loss of silica and the silane coupling agent during mixing with rubber due to the small mixer volume. The mixing procedure is shown in Table 2.

### 2.3. Measurements

A moving die rheometer (MDR) was used to measure the torque values and optimal cure time (*t*_90_) of the uncured rubber compounds at a vibration angle of ±1° and measurement temperatures of 150, 160, and 170 °C. Subsequently, vulcanizates were prepared by pressing for the optimum cure time (*t*_90_) measured at temperatures of 150, 160, and 170 °C. A universal testing machine (UTM, KSU-05M-C, KSU Co., Ansan, Korea) was used to measure the tensile properties of the compounds, including the modulus, tensile strength, and elongation at break. The experimental data from three specimens were averaged. The toughness of the compounds was determined as the area under the stress–strain curves. In accordance with ASTM D412, a 500 N load cell was operated at a speed of 500 mm/min. An ARES-G2 (TA Instruments, New Castle, DE, USA) was used to measure the dynamic viscoelasticity. The test conditions for the measurements were set at an amplitude of 0.5% strain and a frequency of 10 Hz with the torsion mode. The test temperature ranged from −60 °C to 60 °C.

### 2.4. Analysis of Vulcanizate Structure

**Figure 2:**
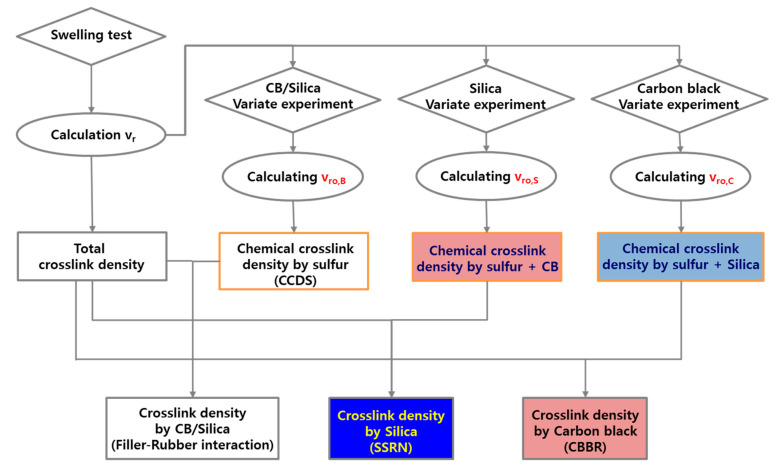
Flowchart of the analysis on vulcanizate structures using the swelling test and Flory–Rehner equation.

The swelling of the cured specimens in toluene was used to measure the crosslink density and vulcanizate structure. The cured specimens were prepared with dimensions of 10 mm × 10 mm × 2 mm. To remove the organic materials, the specimens were immersed in 30 mL of tetrahydrofuran (THF) and n-hexane for 2 days at 25 °C. After removal of the organic materials, the specimens were dried for 3 days in a vacuum oven at 40 °C. To calculate the crosslink density, the dry specimens were immersed in toluene for 24 h at 25 °C to facilitate swelling, and the weight before and after swelling was measured. The vulcanizate structure was determined by calculating the crosslink density using the data from the swelling test, Equations (1) and (2). The value of the volume fraction of rubber in the swollen gel (*V*_1_) in the Flory–Rehner equation was calculated using Equation (3).

(3) $$v_{r}=\frac{\frac{w_{dry}-w_{filler}}{\rho_{rubber}}}{\begin{array}{c} \frac{w_{dry}-w_{filler}}{\rho_{rubber}}+\frac{w_{swollen}-w_{dry}}{\rho_{solvent}} \end{array}}$$

where *w_dry_* is the weight of dry sample, *w_filler_* is the weight of filler in the dry sample, *w_swollen_* is the weight of the swollen sample, *ρ_rubber_* is the density of the rubber, and *ρ_solvent_* is the density of the solvent. According to Boonstra and Kraus et al. [18,19], the ratio volume fraction of rubber in the swollen unfilled rubber to that of the filled rubber ($v_{r0}/v_{r}$) decreases linearly as the filler loading increases. This is due to the different swelling ratios caused by the filler–rubber interaction. This means that the *m* value from Equation (2) suggested by Kraus is an indicator of the filler–rubber interaction. Moreover, substituting *ν_r_* into Equation (1) gives the total crosslink density, and the chemical crosslink density, or the crosslink density of the unfilled vulcanizate, was obtained by substituting *ν_r0_*. Thus, the contributions of the filler–rubber interaction and chemical crosslink density to the total crosslink density were separated quantitatively. Lee et al. conducted swelling tests and used Equations (1) and (2) for vulcanizates filled with silica or carbon black in order to separate the total crosslink density into the filler–rubber interaction and chemical crosslink density [21]. In addition, a previous study by Kim et al. categorized SBR compounds with carbon black and silica as binary fillers into CBBR, SSRN, and CCDS by employing the swelling test as well as Equations (1) and (2), as demonstrated in Figure 2 [25]. In this study, we determined the effect of cure temperature on the vulcanizate structure.

## 3. Results and Discussion

### 3.1. Cure Characteristics

**Figure 3:**
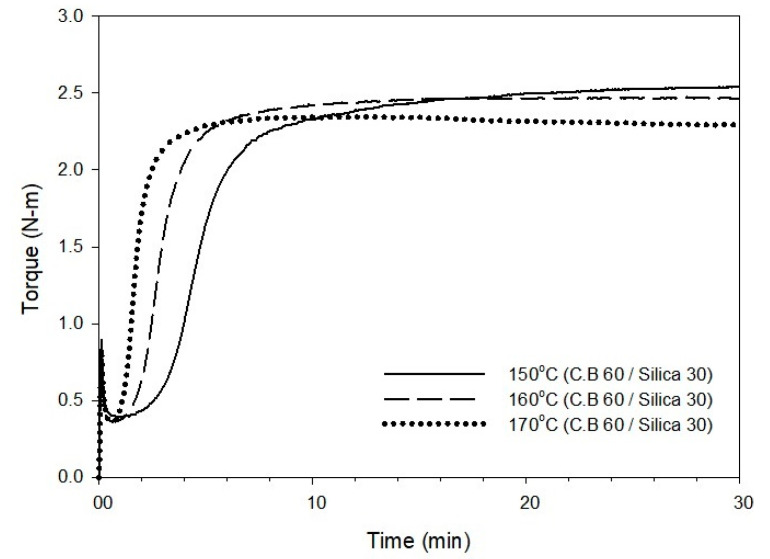
Cure curve of the T-3 compound at different cure temperatures of 150, 160, and 170 °C.

The cure curve and characteristics obtained by varying the cure temperature of the T-3 compound containing 60 phr of carbon black and 30 phr of silica to 150, 160, and 170 °C are shown in Figure 3 and Table 3, respectively. As the cure temperature increases from 150 to 170 °C, the cure time, *t*_90_, decreases by approximately 6 min, and this reduction in cure time can be expected to increase the productivity of the manufacturing process. However, it was shown that there was a roughly 2 min decrease in scorch time, which is a measure that predicts the occurrence of scorch during manufacturing processes, such as extrusion and calendaring. In general, a *t*_10_ value greater than 1 min is favorable for suppressing unwanted scorch, but it is critical to have a prolonged *t*_10_ for scorch safety. Based on the results of the maximum torque (*T*_max_) and Δ*T* (*T*_max_ − *T*_min_) values that predict the total crosslink density with respect to cure temperature, both *T*_max_ and Δ*T* showed a decreasing trend, which suggests a decrease in the crosslink density with an increase in the cure temperature.

**Figure 4:**
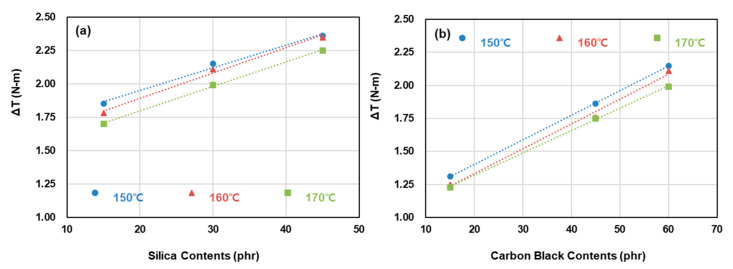
Cure characteristics of compounds with a carbon black and silica binary filler system: (**a**) carbon black contents: 60 phr, silica contents: 15, 30, and 45 phr, (**b**) silica contents: 30 phr, carbon black contents: 15, 45, and 60 phr.

Figure 4 presents Δ*T* as a function of filler content for each cure temperature. Regardless of the filler type, Δ*T* decreased as the cure temperature increased, which is attributed to the decrease in total crosslink density resulting from the decrease in CBBR, SSRN, and CCDS. The crosslink density of each vulcanizate structure is discussed in further detail in Section 3.2. In addition, the values of Δ*T* increased as the contents of silica and carbon black increased, and this result is attributed to the increase in total crosslink density. At low cure temperatures, Δ*T* showed an increase owing to the marching phenomenon, in which the value of *T*_max_ shows a progressive increase. In contrast, a flatter cure curve was obtained as the cure temperature increased, but a further increase in temperature is expected to result in a reversion characterized by a decrease in the *T*_max_ value as the sulfide group is destroyed [9,10]. This phenomenon was observed in the behavior of all compounds (T-1 through T-7) investigated in this study. To obtain the optimal crosslink density and to prevent change in the mechanical properties due to reversion, the mechanical properties and viscoelastic properties of the samples crosslinked at a cure time of *t*_90_ were analyzed.

### 3.2. Analysis of Vulcanizates

**Figure 5:**
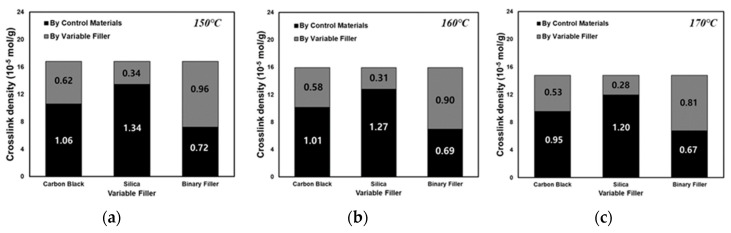
Analysis of the vulcanizate structures of the T-3 compound according to filler variates at different cure temperatures of (**a**) 150, (**b**) 160, and (**c**) 170 °C.

**Figure 6:**
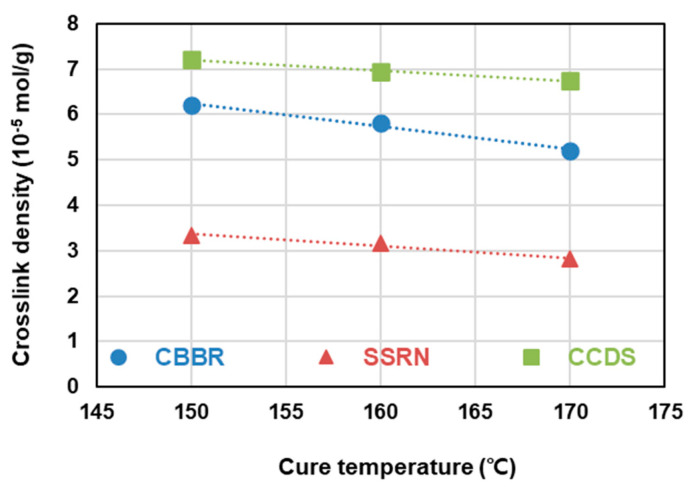
The decreasing rate of the crosslink density of the SBR binary filler compounds according to the cure temperatures.

To analyze the vulcanizate structure, compounds with different filler contents can be used to isolate the sum of the filler–rubber interaction within the total crosslink density resulting from the varied filler, the filler–rubber interaction resulting from the non-varied filler, and the chemical crosslink density caused by sulfur. In this study, the dependence of the vulcanizate structure on the cure temperature was investigated by varying the filler content in compounds T-1 through T-7 and crosslinking them at cure temperatures of 150, 160, and 170 °C. The results of the vulcanizate structure analysis are shown in Table 4 and Figure 5. In accordance with the scheme presented in Figure 2, the sum of the CBBR value (6.21 × 10^−5^ mol/g) calculated from compounds T-1, T-2, and T-3 crosslinked at 150 °C by varying the carbon black content at a fixed silica content of 30 phr and the SSRN value (3.35 × 10^−5^ mol/g) calculated from compounds T-3, T-4, and T-5 crosslinked at 150 °C by varying the silica content at a fixed carbon black content of 60 phr was consistent with the filler–rubber interaction value (9.6 × 10^−5^ mol/g) calculated from compounds T-3, T-6, and T-7 containing varying contents of silica and carbon black. Based on these findings, we verified that the vulcanizate structures can be categorized into CBBR, SSRN, and CCDS in an SBR binary filler system. Vulcanizate structures at 160 °C and 170 °C were determined using the same method, and the crosslink density of each vulcanizate structure and its decrease rate are shown in Table 4 and Figure 6. As the cure temperature increased, CBBR, SSRN, and CCDS all decreased, and the total crosslink density also decreased. CBBR showed the largest decrease, which is attributed to a more significant breaking of physical bound rubber in CBBR, relative to the chemical networks of SSRN and CCDS. Moreover, it can be seen that the ratio of CCDS to the total crosslink density gradually increases owing to the fast decrease in CBBR.

To determine the effect of the filler–rubber interaction due to silica and carbon black on the crosslink density in the vulcanizate structure of the binary filler system, CBBR and SSRN were each divided into the carbon black and silica contents to calculate the values per 1 phr of filler contributing to the crosslink density. Regardless of the cure temperature, 1 phr of silica had a greater effect on the crosslink density, as shown in Table 5. This is believed to have resulted from the difference between the specific surface areas of silica (BET: 175 m^2^/g) and carbon black (BET: 114 m^2^/g) [26]. In addition, the decrease in CBBR/phr was shown to be greater than that in SSRN/phr with increasing cure temperature. This is due to the physical bound rubber between carbon black and the rubber being more easily destroyed by high temperature, as described earlier.

### 3.3. Mechanical Properties

**Figure 7:**
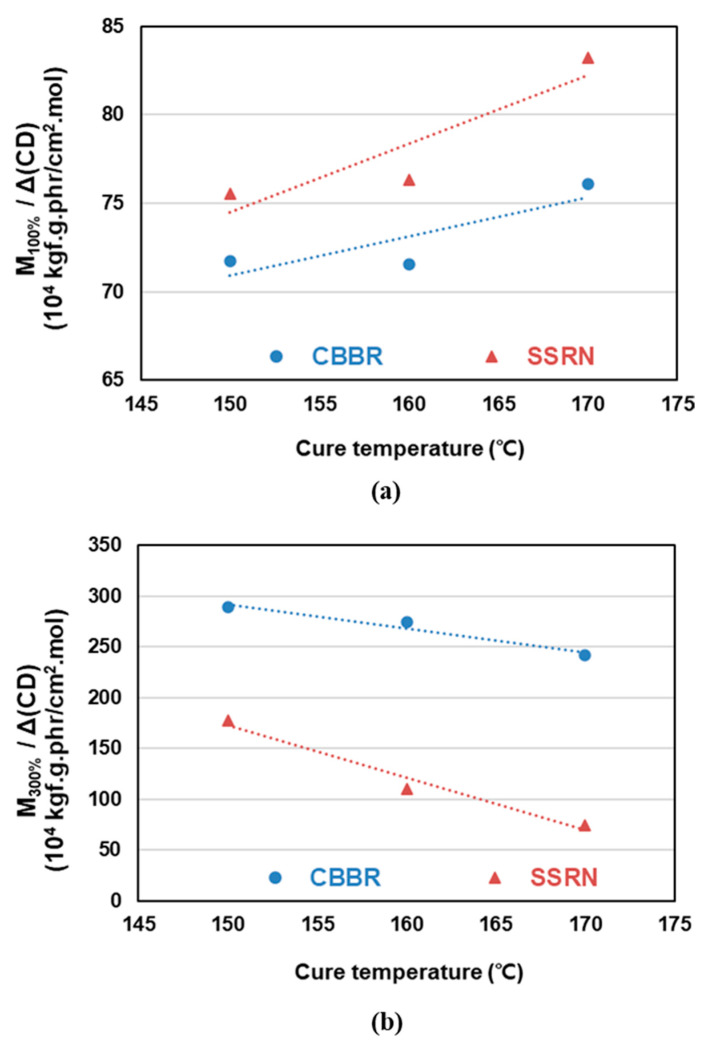
Change of the mechanical properties of the SBR binary filler compounds by different cure temperatures: (**a**) 100% modulus and (**b**) 300% modulus.

**Figure 8:**
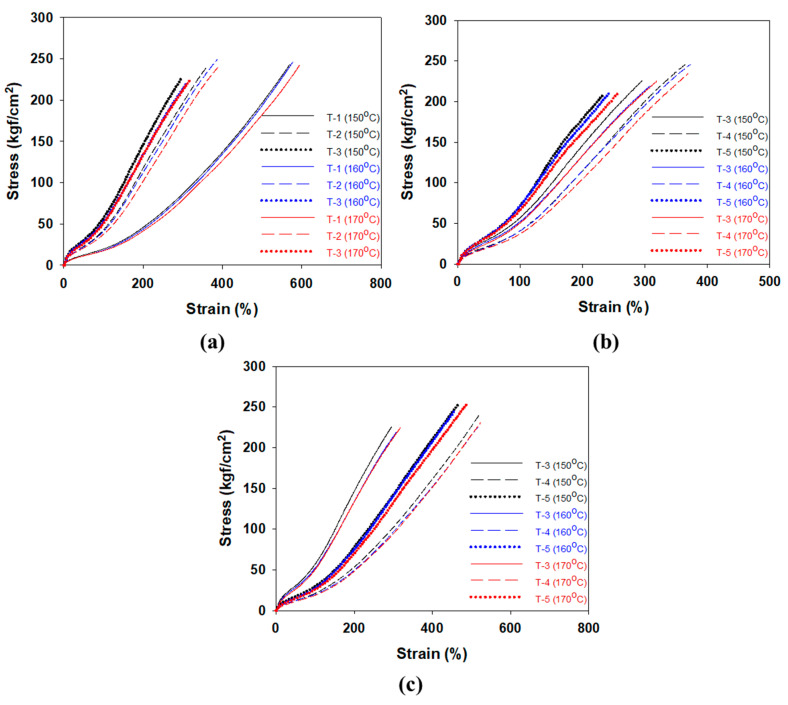
Stress–strain curves of the vulcanizates by different cure temperature; (**a**) T-1,2,3, (**b**) T-3,4,5, and (**c**) T-3,6,7.

The values per 1 phr of filler contributing to the crosslink density at different cure temperatures were calculated in Section 3.2. The effects of CBBR/phr and SSRN/phr (i.e., the contribution of 1 phr of filler to the crosslink density) on the modulus at 100% elongation (*M*_100%_) at different cure temperatures are shown in Table 6 and Figure 7a. Stress–strain curves of the vulcanizates by different cure temperatures are shown in Figure 8. In particular, across all cure temperatures, the effect of SSRN/phr is greater than that of CBBR/phr on *M*_100%_. Moreover, the effects of SSRN/phr and CBBR/phr on *M*_100%_ increased as the cure temperature increased. However, this result is in contrast to the decreasing trends of CBBR, SSRN, and CCDS with increasing cure temperature, as mentioned in Section 3.2. This is due to the increase in the effect of CCDS on the total crosslink density resulting from the significant decrease in the filler–rubber interaction (CBBR+SSRN) with the increase in cure temperature. In other words, the effect of CCDS on *M*_100%_ is greater than that of CBBR and SSRN. Moreover, the effect of each filler on *M*_100%_ was greater for SSRN than for CBBR. This is because of the chemical network between silica and silane, which leads to the formation of a stronger filler–rubber interaction [26].

The effects of CBBR/phr and SSRN/phr on the modulus at 300% elongation (*M*_300%_) at different cure temperatures are presented in Table 6 and Figure 7b. Regardless of the cure temperature, *M*_300%_ (i.e., high strain) is more strongly affected by CBBR/phr than SSRN/phr. This is due to the breakdown of the filler–filler network of silica compounds at high strain [26]. Furthermore, for the carbon black variate, this is attributed to the strong physical interaction between carbon black and rubber at high strain [27]. However, unlike *M*_100%_, the effects of SSRN and CBBR decreased as the cure temperature increased. This is due to the decrease in total crosslink density resulting from the decrease in CBBR, SSRN, and CCDS with the increase in cure temperature. In addition, the magnitude of the rate of change (RoC) of *M*_300%_ with increasing cure temperature was smaller in CBBR than in SSRN. This is due to a more significant effect of CBBR on *M*_300%_ than SSRN [26]. Even though rubber forms a chemical bond through the coupling reaction with silica, the chemical bond becomes weak at a strain of 100% or more. However, the rubber adsorbed to carbon black forms a physical crosslink and exhibits a higher crosslink density due to the glassy bound rubber. In particular, according to the interface model by Y. Fukahori, the bound rubber has a dual-layer structure consisting of an internal polymer layer in a glassy state and a softer external polymer layer [27]. As such, in the high strain range, the rubber molecules of the external polymer layer are oriented in strands along the elongation direction due to slippage and show a large reinforcement effect. In other words, *M*_300%_ (i.e., stress value in the high strain region) is more strongly affected by the filler–rubber network of SSRN and CBBR than by CCDS.

### 3.4. Dynamic Viscoelasticity

**Figure 9:**
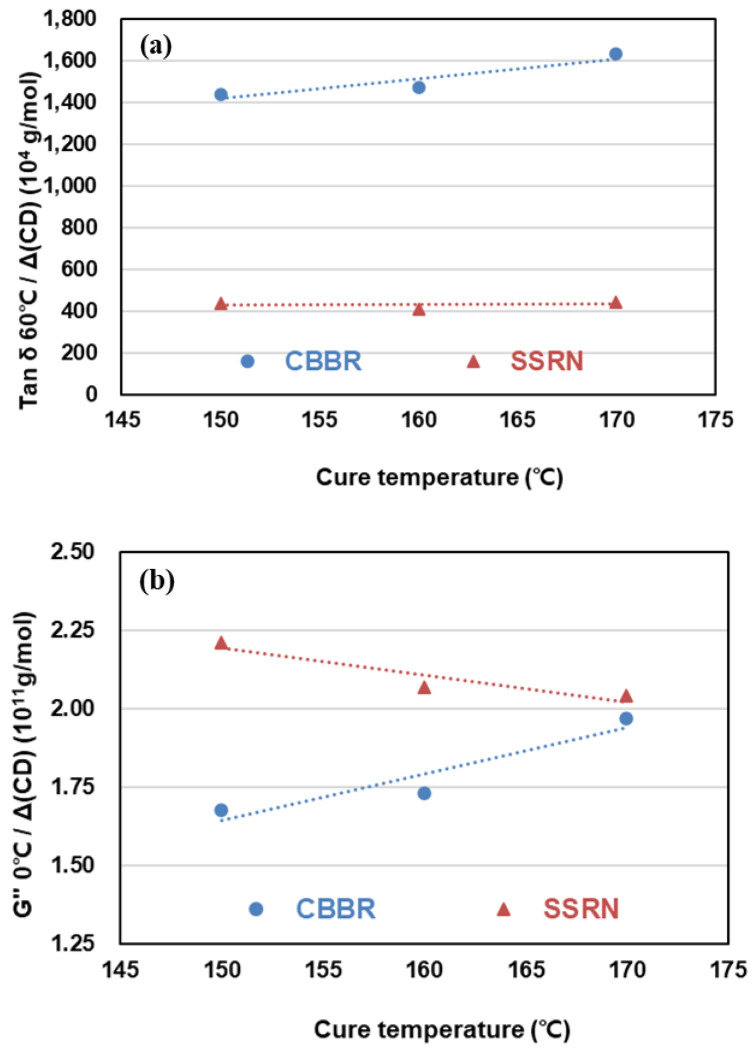

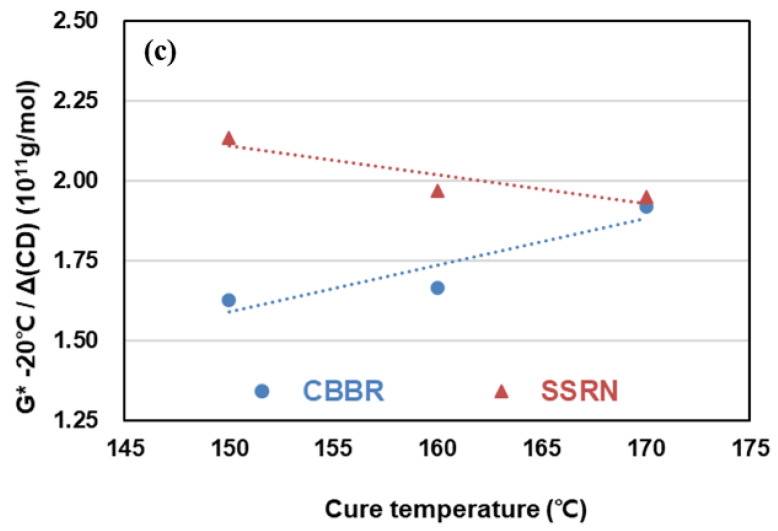
Change of viscoelasticity properties of the SBR binary filler compounds by different cure temperatures: (**a**) tan ẟ at 60 °C, (**b**) *G*″ at 0 °C, and (**c**) G* at −20 °C.

The performance of tire treads and the dynamic viscoelasticity of compounds exhibit an excellent correlation. In particular, the complex modulus (G*) at −20 °C, loss modulus (*G*″) at 0 °C, and tan δ at 60 °C are used as indicators of snow traction, wet traction, and rolling resistance, respectively [28,29,30,31]. The effects of CBBR/phr and SSRN/phr that contributed to the crosslink density per 1 phr of filler with increasing cure temperature on snow traction, wet traction, and rolling resistance (RR) in SBR vulcanizates with binary filler system are shown in Table 7 and Figure 9. It is known that a lower tan δ at 60 °C is favorable for RR [32,33]. Curing at a high temperature (170 °C) resulted in a small increase in tan δ at 60 °C due to SSRN and showed a positive effect on RR. However, the tan δ at 60 °C due to CBBR increased and showed a negative effect on RR, and a higher cure temperature led to a larger increase in the value. As described in Section 3.2, this is attributed to the decrease in total crosslink density resulting from the decrease in CBBR, SSRN, and CCDS with the increase of cure temperature. The effect of CBBR/phr and SSRN/phr on the RoC of tan δ at 60 °C is shown in Table 7. In particular, the RoC of tan δ at 60 °C by CBBR is greater than that by SSRN with increasing cure temperature. As mentioned in Section 3.2, this is attributed to the larger RoC of the crosslink density due to CBBR than SSRN, which is because the bound rubber (i.e., the physical network between carbon black and rubber) is more easily destroyed by the high cure temperature than the chemical networks of SSRN and CCDS.

The crosslink density contribution from 1 phr of filler to *G*″ at 0 °C and the rate of change of *G*″ at 0 °C, which can predict wet traction, were investigated with respect to the cure temperature. At all cure temperatures, SSRN/phr showed higher values of *G*″ at 0 °C than CBBR/phr. Among the viscoelastic properties, a higher value of *G*″ at 0 °C is known to be advantageous for wet traction. In this study, SSRN/phr compared to CBBR/phr was shown to have a more significant effect on wet traction in terms of the contribution of the crosslink density per unit content of filler. The rate of change of *G*″ at 0 °C with respect to cure temperature showed opposite trends in SSRN and CBBR. This is shown in Figure 9b, which demonstrates that the influence of SSRN on *G*″ at 0 °C decreased and that of CBBR increased as cure temperature increased. Moreover, the magnitude of the RoC of *G*″ at 0 °C by CBBR was greater than that by SSRN. It can be concluded that the influence of filler properties is more significant than that of the vulcanizate structure due to the presence of carbon black and silica with respect to cure temperature.

It is known that a lower value of G* at −20 °C is more desirable for snow traction [28]. Regardless of the cure temperature, SSRN/phr compared to CBBR/phr resulted in higher values of G* at −20 °C. In addition, the effect of SSRN on G* at −20 °C increased and that of CBBR decreased as cure temperature increased. That is, CBBR/phr can be seen to have a more significant influence on snow traction.

The effects of SSRN and CBBR on the RoC of G* at −20 °C exhibited opposite trends, as shown in Table 7. Furthermore, it can be seen that the magnitude of the RoC of G* at −20 °C by CBBR is greater than that of SSRN. This is in agreement with the results on how *G*″ at 0 °C is affected by each filler, and the same trends seen in wet traction and snow traction are consistent with previous research findings by Kim et al. [26].

## 4. Conclusions

In this study, vulcanizate structures at different cure temperatures were categorized into CBBR, SSRN, and CCDS and quantified. The effect of each vulcanizate structure at different cure temperatures on the mechanical and viscoelastic properties was also investigated. CBBR is composed of a physical network between carbon black and rubber, while SSRN and CCDS consist of chemical networks. The analysis of the vulcanizate structures revealed that CBBR, SSRN, and CCDS all showed decreasing trends with increasing cure temperature, and CBBR resulted in the largest rate of decrease.

Analysis of the contribution of 1 phr of filler content to the filler–rubber interaction showed that silica (SSRN/phr) had a greater effect than carbon black (CBBR/phr), and this result was attributed to the difference between the specific surface areas of silica (BET: 175 m^2^/g) and carbon black (BET: 114 m^2^/g) [26]. The cure analysis revealed that Δ*T* (*T*_max_ − *T*_min_) decreased as cure temperature increased, regardless of the filler type. This was considered to be the result of the decrease in the total crosslink density due to the decrease in CBBR, SSRN, and CCDS with the increase in cure temperature at the SBR binary filler system. Investigation of the mechanical properties demonstrated that the 100% modulus was strongly affected by CCDS, while the 300% modulus was more significantly affected by the filler–rubber networks of SSRN and CBBR than CCDS.

Analysis of the viscoelastic properties showed that the influence of SSRN and CBBR on tan δ at 60 °C at different cure temperatures had an excellent correlation with the vulcanizate structure analysis results. The absolute effect of SSRN on tan δ at 60 °C was superior to that of CBBR. However, owing to the greater rate of decrease in the filler–rubber interaction of CBBR than that of SSRN with an increase in cure temperature, CBBR resulted in a larger increase in the value of tan δ at 60 °C. Therefore, it can be seen that the improvement of the rolling resistance of compounds using silica as the filler cured at low temperatures is lower than that in compounds using carbon black as the filler. On the other hand, the effects of SSRN and CBBR on both *G*″ at 0 °C and G* at −20 °C showed opposite trends as the cure temperature increased. This is ascribed to the stronger influence of the inherent properties of the filler compared to the changes in CBBR and SSRN with respect to cure temperature.

As such, through the analysis of the vulcanizate structures at different cure temperatures in a binary filler system (carbon black/silica), this study aims to present a guide for effectively increasing the crosslink density of rubber and for showing the necessary mechanical and viscoelastic properties. The performance changes in the mechanical properties of the compounds with respect to the cure temperature and carbon black/silica ratio are summarized in Table 8. The results presented here can serve as an important guideline for determining the content and type of filler and for setting the cure condition during the design of actual compound formulations.

## Figures and Tables

**Table 1:** Experimental formulations for compounds with carbon black and silica binary fillers given in parts per hundred rubber (phr).

Compound Code		T-1	T-2	T-3	T-4	T-5	T-6	T-7
Stage 1	ESBR	100	100	100	100	100	100	100
	Carbon black (N110)	15	45	60	60	60	30	40
	Silica (7000GR)	30	30	30	15	45	15	20
	X50-S *	4.8	4.8	4.8	2.4	7.2	2.4	3.2
	TDAE oil	13.3	13.3	13.3	13.3	13.3	13.3	13.3
	Zinc oxide	3	3	3	3	3	3	3
	Stearic acid	2	2	2	2	2	2	2
	6PPD	2	2	2	2	2	2	2
	TMQ	2	2	2	2	2	2	2
Stage 2	Stage 1 masterbatch	200	200	200	200	200	200	200
	Sulfur	1.6	1.6	1.6	1.6	1.6	1.6	1.6
	DPG	0.5	0.5	0.5	0.5	0.5	0.5	0.5
	TBBS	2.6	2.6	2.6	2.6	2.6	2.6	2.6
	PVI	0.2	0.2	0.2	0.2	0.2	0.2	0.2

* The amounts of the silane coupling agents were calculated as 16 wt% of the weight of silica.

**Table 2:** Compounding procedure for the styrene butadiene rubber (SBR) binary filler compounds.

Stage 1	
0–20″	Rubbers (initial temperature: 110 °C)
20″–1′00	1/2 filler (carbon black)
1′00″–1′40″	1/2 filler (carbon black)
1′40″–2′40″	Oil
2′40″–3′40″	1/2 filler (silica), 1/2 silane
3′40″–4′40″	1/2 filler (silica), 1/2 silane
4′40″–5′40″	ZnO, Stearic acid, 6PPD, TMQ
5′40″–10′40″	Extra mix and dump (dump temperature: 150–155 °C)
**Stage 2**	
0–20″	Compounds of stage 1 (initial temperature: 50 °C)
20″–1′	Sulfur, cure accelerators (TBBS, DPG, PVI)
1′–2′	Extra mix and dump (dump temperature: 100 °C))

**Table 3:** Cure characteristics of the T-3 compound at different cure temperatures.

Cure Temperature	150 °C	160 °C	170 °C
Filler contents (C.B/Silica)	60/30		
*t_10_*/*t_90_* (min:sec)	03:05/09:54	01:53/05:12	01:11/03:05
*T*_min_/*T*_max_ (N·m)	0.390/2.553	0.363/2.473	0.355/2.346
Δ*T* (*T*_max_ − *T*_min_, N·m)	2.15	2.11	1.99

**Table 4:** Vulcanizate structure of the SBR binary compound (T-3) at different cure temperatures.

Crosslink Density (10^−5^ mol/g)	150 °C	160 °C	170 °C	Decrease Rate (10^−5^ mol/g∙°C)
CBBR	6.21 [37.0%]	5.82 (−0.39) [36.5%]	5.21 (−0.61) [35.2%]	−0.050
SSRN	3.35 [20.0%]	3.17 (−0.18) [19.9%]	2.82 (−0.35) [19.1%]	−0.027
CCDS	7.22 [43.0%]	6.95 (−0.27) [43.6%]	6.75 (−0.20) [45.7%]	−0.024
Total	16.78	15.94 (−0.84)	14.78 (−1.16)	−0.101

**Table 5:** Crosslink density per one phr of filler by different cure temperatures.

Crosslink Density per phr of Filler(10^−6^ mol/g∙phr)	150 °C	160 °C	170 °C	Decrease Rate (10^−6^ mol/g∙°C)
CBBR/phr	1.16	1.08	0.96	−0.0098
SSRN/phr	1.32	1.27	1.15	−0.0085

**Table 6:** Mechanical properties per one phr of filler by different cure temperatures.

Description	*M*_100%_ per CBBR or SSRN (10^4^ kgf·g·phr/cm^2^·mol)				*M_300%_* per CBBR or SSRN (10^4^ kgf·g·phr/cm^2^·mol)			
Cure Temperature	150 °C	160 °C	170 °C	RoC *	150 °C	160 °C	170 °C	RoC *
CBBR	71.71	71.57	76.09	0.219	288.85	274.26	241.62	−2.362
SSRN	75.54	76.32	83.24	0.385	178.03	110.43	74.92	−5.156

* RoC: Rate of Change; 10^4^ kgf·g·phr/cm^2^·mol °C.

**Table 7:** Viscoelasticity properties per one phr of filler by different cure temperatures.

Cure Temperature		150 °C	160 °C	170 °C	RoC
Tan ẟ at 60 °C per CBBR or SSRN (10^4^ g∙phr/mol)	Carbon black variate (CBBR)	1439.7	1468	1628.9	9.46
	Silica variate (SSRN)	439.14	411.29	442.43	0.165
*G*″ at 0 °C per CBBR or SSRN (10^11^ MPa·g∙phr/mol)	Carbon black variate (CBBR)	1.676	1.732	1.971	0.015
	Silica variate (SSRN)	2.213	2.071	2.041	-0.008
G* at −20 °C per CBBR or SSRN (10^11^ MPa·g∙phr/mol)	Carbon black variate (CBBR)	1.626	1.667	1.917	0.145
	Silica variate (SSRN)	2.135	1.970	1.951	−0.092

**Table 8:** Effect of silica-silane–rubber network (SSRN) or carbon black bound rubber (CBBR) on the performance change of rubber compounds with increasing cure temperature.

Item	Cure Temp.	Crosslink Density	M_100%_	M_300%_	Fuel Economy	Wet Traction	Snow Traction
Increase of CB ratio	Low (150 °C)	G	G	E	B	B	G
	High (170 °C)	B	B	G	W	M	B
Increase of Silica ratio	Low (150 °C)	E	E	G	E	G	W
	High (170 °C)	M	G	M	G	M	B

E: excellent; G: good; M: moderate; B: bad; W: worst.
